# Supplementary material for: An assembly of nuclear bodies associates with the active VSG expression site in African trypanosomes
Source: Nat Commun. 2022 Jan 10;13:101. doi: 10.1038/s41467-021-27625-6 (PMC8748868; doi:10.1038/s41467-021-27625-6)
Supplement: Supplementary file 1 — Supplementary Information File [file 41467_2021_27625_MOESM1_ESM.pdf]

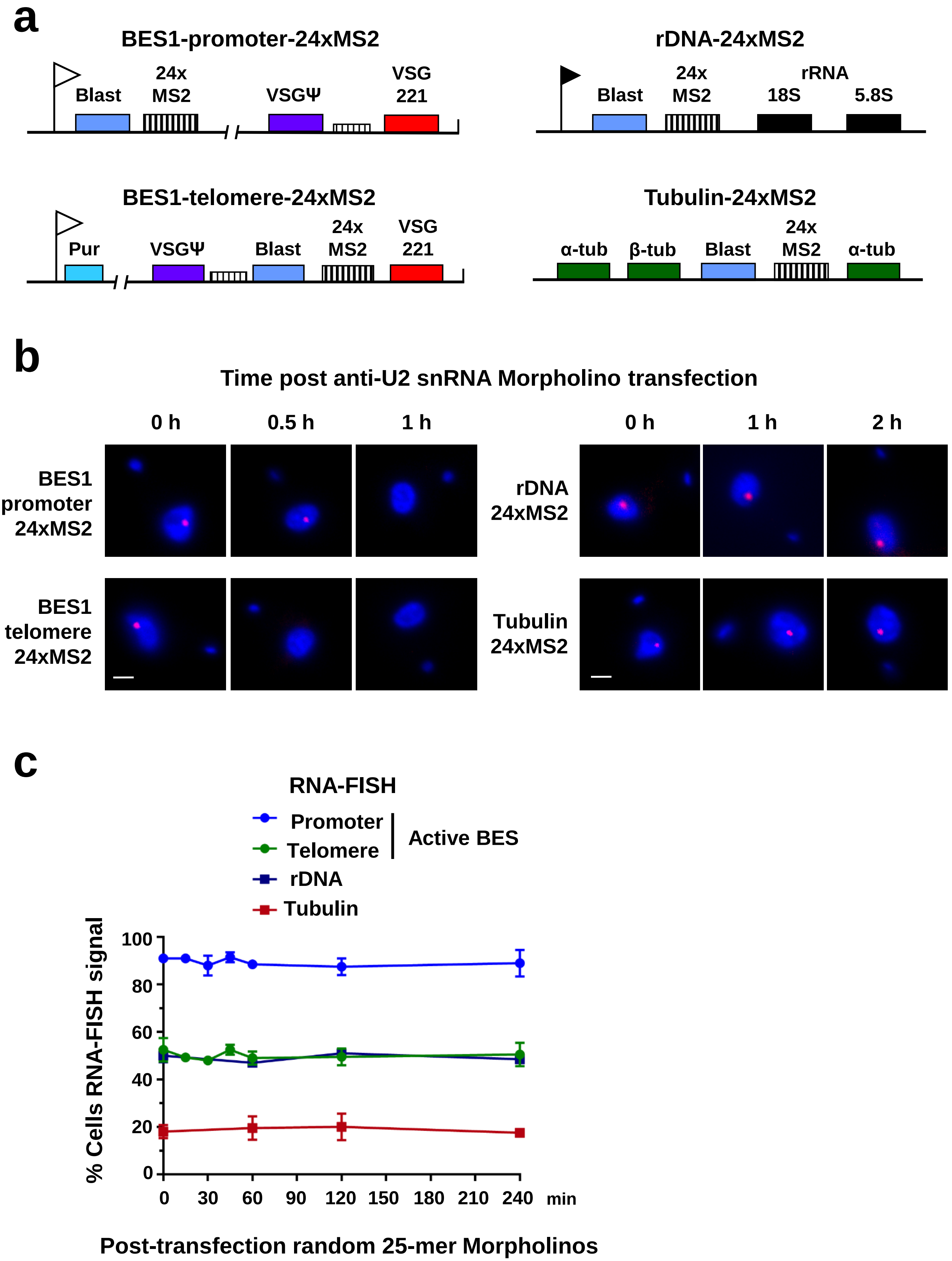
a
BES1-promoter-24xMS2
Blast
24x MS2
VSGΨ
VSG 221
rDNA-24xMS2
Blast
24x MS2
rRNA
18S
5.8S
BES1-telomere-24xMS2
Pur
VSGΨ
Blast
24x MS2
VSG 221
Tubulin-24xMS2
α-tub
β-tub
Blast
24x MS2
α-tub
b
Time post anti-U2 snRNA Morpholino transfection
0 h
0.5 h
1 h
0 h
1 h
2 h
BES1 promoter 24xMS2
rDNA 24xMS2
BES1 telomere 24xMS2
Tubulin 24xMS2
c
RNA-FISH
Promoter
Telomere
Active BES
rDNA
Tubulin
% Cells RNA-FISH signal
100
80
60
40
20
0
0
30
60
90
120
150
180
210
240
min
Post-transfection random 25-mer Morpholinos

Supplementary Figure 1

a) Schematic diagrams of the genomic loci (not to scale) marked with constructs containing 24x MS2 sequence repeats (vertically hatched boxes) flanked by a blasticidin (blast) resistance gene. The 221 ES (BES1) contains a VSG pseudogene (VSGΨ) near to the telomeric VSG221 gene, as well as a puromycin (Pur) resistance gene. Promoters are indicated with flags, and characteristic 70 bp repeats with small hatched boxes. The ribosomal DNA (rDNA) locus contains rRNA genes (black boxes) and a promoter (black flag). The tubulin locus contains alternating α- and β-tubulin (tub) genes (green boxes).

b) Representative fluorescence microscopy images of the cell lines shown in **a)** using RNA-FISH detecting nascent MS2 repeat containing transcript (red), after the inhibition of splicing through transfection of the cells with anti-U2 snRNA Morpholinos. Time after transfection is indicated above in hours (h). DNA is stained with DAPI (blue) and scale bars = 1 μm. Quantitation of these results are shown in Fig. 1 d).

c) Quantitation of the percentage (%) of cells with MS2 RNA-FISH signal in cell lines with different genomic loci marked as described in **a**), observed after the time indicated in minutes (min) after transfection with random 25-mer Morpholinos. Minimally 300 cells in G1 (1K1N) were counted for each time point. Values are the averages of three biological replicates with error bars indicating ± SD.

Source data are provided as a Source Data file.

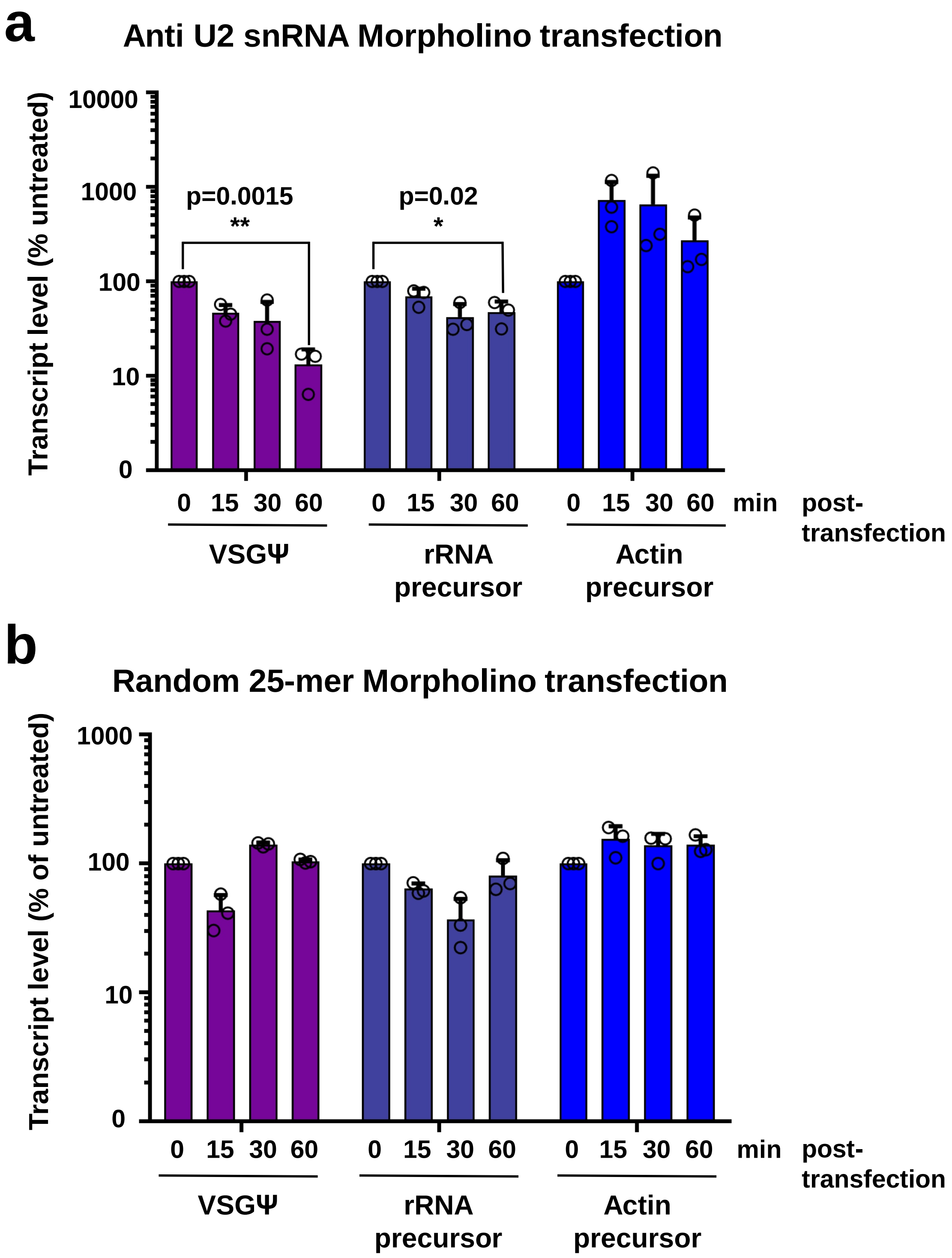
a
Anti U2 snRNA Morpholino transfection
Transcript level (% untreated)
10000
1000
100
10
0
p=0.0015
**
p=0.02
*
0 15 30 60
0 15 30 60
0 15 30 60
min
post-
transfection
VSGΨ
rRNA
precursor
Actin
precursor
b
Random 25-mer Morpholino transfection
Transcript level (% of untreated)
1000
100
10
0
0 15 30 60
0 15 30 60
0 15 30 60
min
post-
transfection
VSGΨ
rRNA
precursor
Actin
precursor

Supplementary Figure 2

Inhibition of splicing through transfection of anti U2 snRNA Morpholinos disrupts RNA production from the active ES.

- **a)** Quantitation of precursor RNA from the active BES1 expressing VSG221 (VSG pseudogene, VSGΨ), rDNA or actin loci using the cell lines described in Supplementary Fig. 1a. RNA was quantitated using qPCR after transfection of cells with anti-U2 snRNA Morpholinos for the time indicated in minutes (min). Transcripts were normalised to the 0 minute time point. Average values are shown from three biological replicates with error bars indicating ± SD. The statistical significance is indicated above. P values were determined using a two tailed paired Student's t-test. For the difference in transcript levels after 0 min versus 60 min timepoints, P=0.0015 and P=0.02 for VSGΨ and rRNA precursor, respectively.
- **b)** As in **a)** only random 25-mer Morpholinos were used in the transfection instead of Morpholinos specific for U2 snRNA.

The values from individual replicates are shown with circles. Source data are provided as a Source Data file.

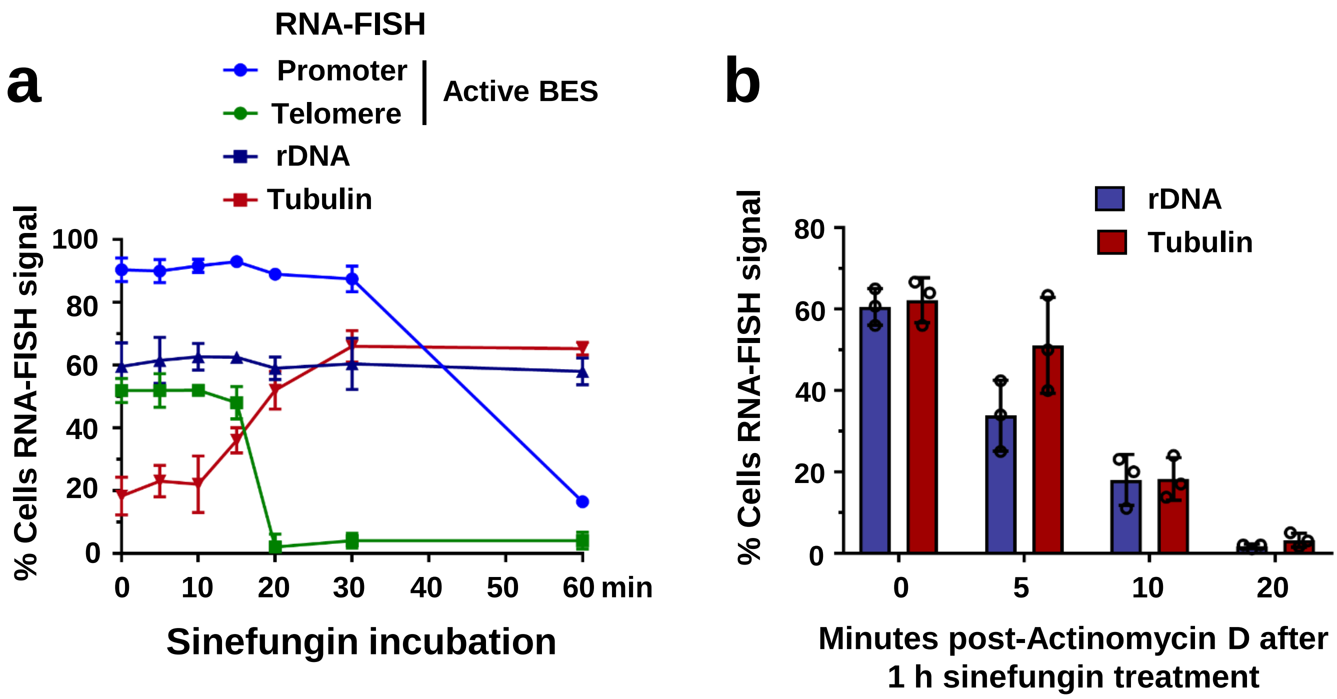
a
RNA-FISH
Promoter
Telomere
Active BES
rDNA
Tubulin
% Cells RNA-FISH signal
100
80
60
40
20
0
0
10
20
30
40
50
60 min
Sinefungin incubation
b
rDNA
Tubulin
% Cells RNA-FISH signal
80
60
40
20
0
0
5
10
20
Minutes post-Actinomycin D after
1 h sinefungin treatment

Supplementary Figure 3

Inhibition of *trans*-splicing using sinefungin results in a block in transcriptional elongation at the active ES.

a) Quantitation of the percentage (%) of cells with MS2 RNA-FISH signal after treatment with 2.5 µg ml^{-1} sinefungin for the time indicated in minutes (min) using the MS2 tagged cell lines shown in Supplementary Fig. 1a. Minimally 200 (1K1N) cells were counted per time point. Values are the averages of three biological replicates with error bars indicating ± SD.

b) Quantitation of the percentage (%) of cells with MS2 RNA-FISH signal from the rDNA or tubulin locus after inhibition of splicing with sinefungin (2.5 µg ml^{-1}) for 1 hour (h) followed by inhibition of transcription with Actinomycin D (10 µg ml^{-1}) for the time indicated in minutes. Minimally 150 cells in G1 (1K1N) were counted per time point. Values shown are the averages of three biological replicates with error bars showing ± SD. The values from individual replicates are shown with circles.

Source data are provided as a Source Data file.

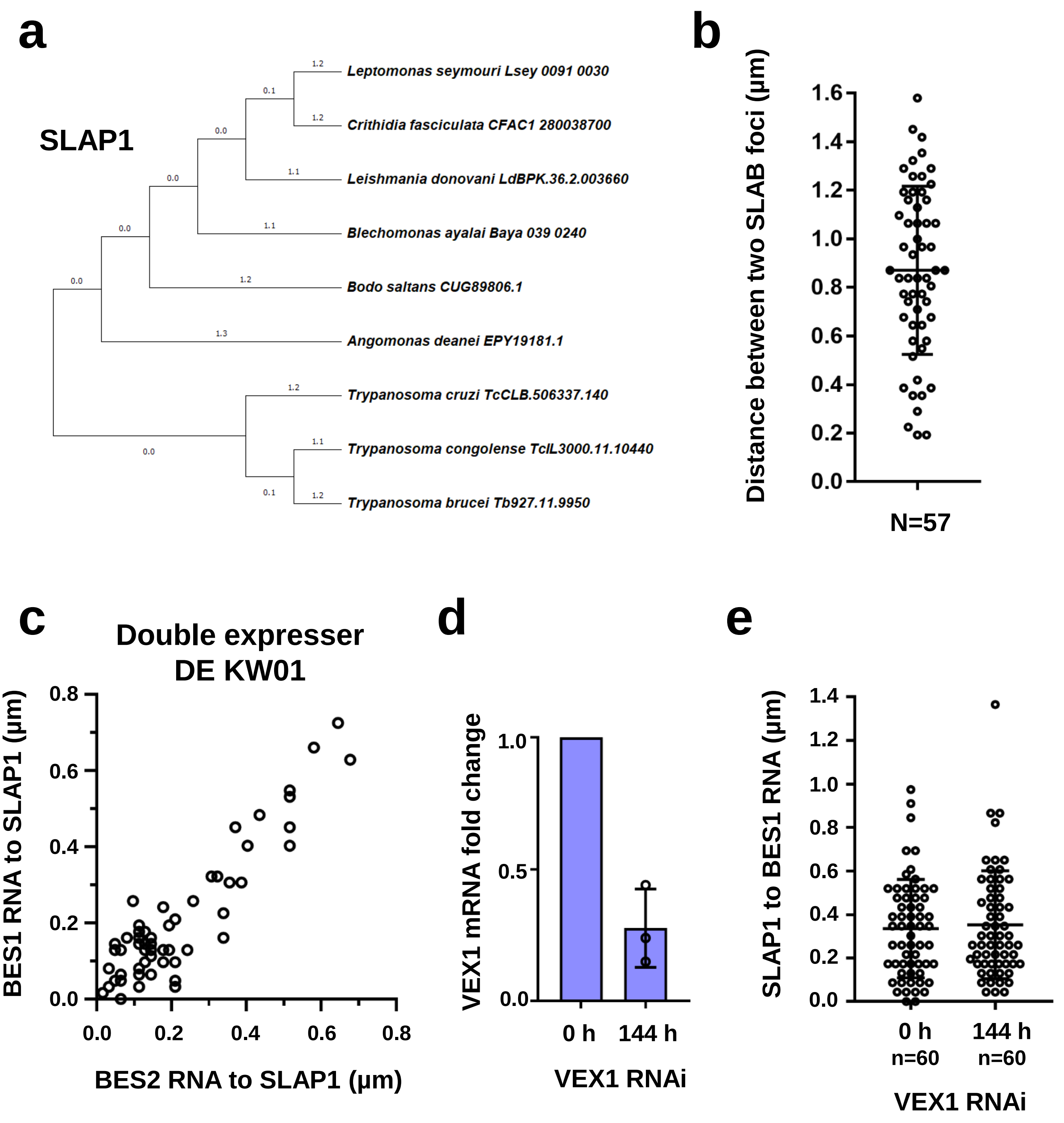
a
SLAP1
Leptomonas seymouri Lsey 0091 0030
Crithidia fasciculata CFAC1 280038700
Leishmania donovani LdBPK.36.2.003660
Blechomonas ayalai Baya 039 0240
Bodo saltans CUG89806.1
Angomonas deanei EPY19181.1
Trypanosoma cruzi TcCLB.506337.140
Trypanosoma congolense TcIL3000.11.10440
Trypanosoma brucei Tb927.11.9950
b
Distance between two SLAB foci (µm)
N=57
c
Double expresser
DE KW01
BES1 RNA to SLAP1 (µm)
BES2 RNA to SLAP1 (µm)
d
VEX1 mRNA fold change
0 h
144 h
VEX1 RNAi
e
SLAP1 to BES1 RNA (µm)
0 h
n=60
144 h
n=60
VEX1 RNAi

Supplementary Figure 4

Analysis of the SLAB focus which associates with the active ES in a VEX1 independent manner.

- **a)** Phylogenetic tree of SLAP1 homologues in other Kinetoplastid protozoa created with MEGA-X using the neighbour joining method. Homologues were found by searching the pBLAST database with the *T. brucei* SLAP1 (Tb927.11.9950) CDS. Hits were considered significant when $e<0.005$. Branch lengths are drawn to scale and represent the number of substitutions per sequence site.
- **b)** Quantitation of the distance between SLAB foci in cells where two SLAB foci were visible within the nucleus. Data were collected from two biological replicates with error bars indicating ± SD.
- **c)** Quantitation of the distance from BES1 or BES2 nascent RNA to the SLAB focus (SLAP1) using double-expresser DE KW01-MS2-V02 cells (shown in Fig. 2f) in which RNA from either BES1 or BES2 individually is detectable. N=53 cells in G1 (1K1N) from two biological replicates.
- **d)** Quantitation of VEX1 mRNA levels using qRT-PCR with RNA from the cell line used in e) after induction of VEX1 RNAi for the time indicated in hours (h). Transcript levels were normalised to actin. Average values are shown from three biological replicates, with error bars indicating ± SD.
- **e)** Quantitation of the distance from the SLAB (SLAP1) to the active BES1 in the presence of VEX1 RNAi for the time indicated in hours (h). The cell line used was BF *T. brucei* expressing mNG::SLAP1, with RNA-FISH detecting nascent transcript from the active BES1 using a VSG pseudogene (VSGΨ) probe. Values shown are the averages from two biological replicates with error bars indicating ± SD, and the number of cells counted (n) indicated below.

Source data are provided as a Source Data file.

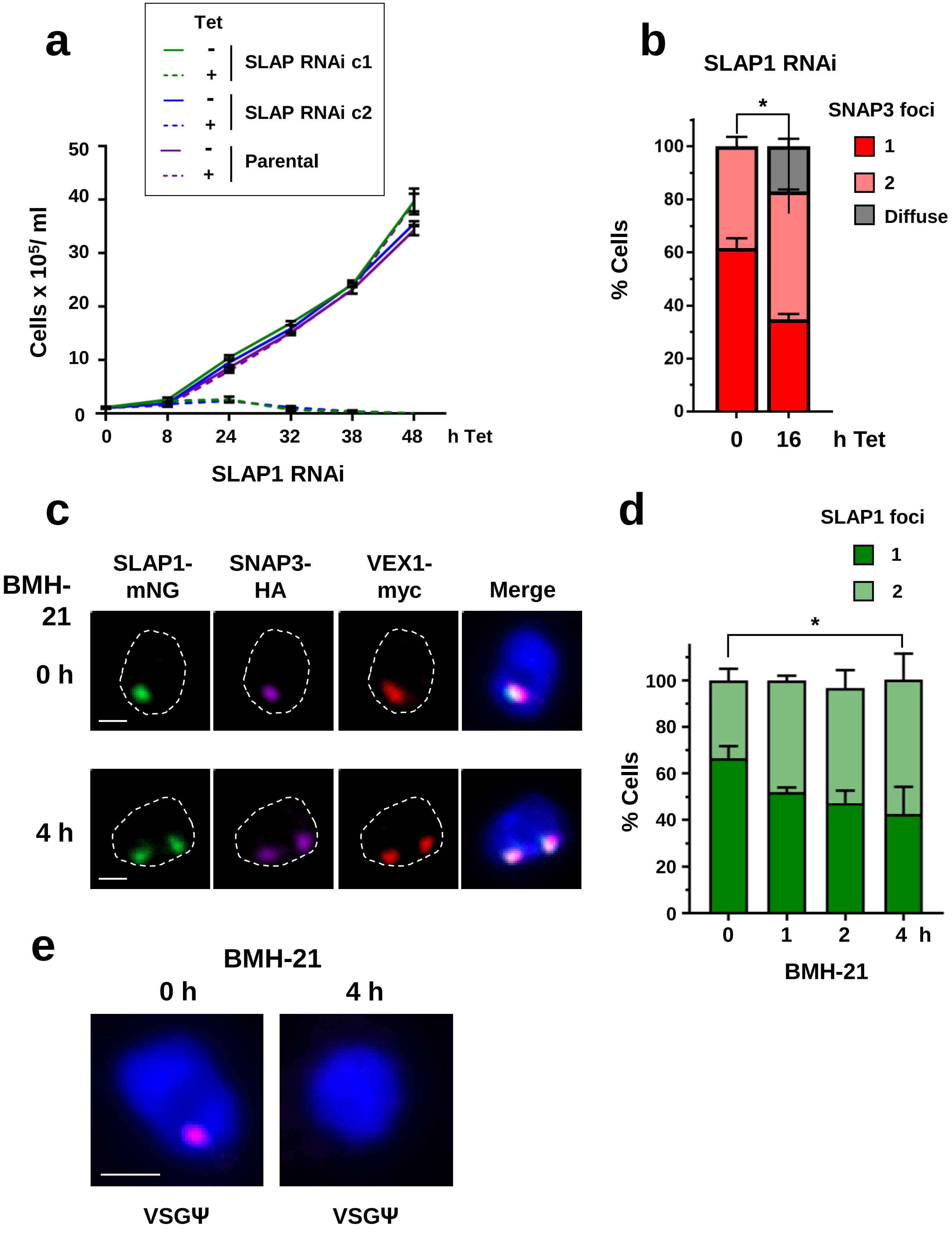
a
Tet
-
+
SLAP RNAi c1
-
+
SLAP RNAi c2
-
+
Parental
Cells x 10⁵/ ml
h Tet
SLAP1 RNAi
b
SLAP1 RNAi
SNAP3 foci
1
2
Diffuse
% Cells
*
h Tet
c
BMH-21
SLAP1-mNG
SNAP3-HA
VEX1-myc
Merge
0 h
4 h
d
SLAP1 foci
1
2
% Cells
*
h
BMH-21
e
BMH-21
0 h
4 h
VSGΨ
VSGΨ

Supplementary Figure 5

The SLAB is essential for cell viability and is partially disrupted by RNA Pol I transcription inhibition.

- **a)** Growth curves of BF *T. brucei* cell lines where SLAP1 RNAi was induced in the presence (+) or absence (-) of tetracycline (Tet), compared with a parental cell line. Cell density was monitored for the time indicated in hours (h) in two independently generated SLAP1 RNAi clones (c1, c2). Values shown are the averages from three technical replicates with error bars indicating ± SD.
- **b)** Quantitation of the percentage (%) of cells with different numbers of SNAP3 foci before or after the induction of SLAP1 RNAi with tetracycline (Tet) for the time indicated in hours (h), using the cell line shown in Fig. 3c. N is equal to minimally 300 1K1N cells for each time point. Values shown are averages from three biological replicates with error bars indicating ±SD. *, P=0.024.
- **c)** Representative immunofluorescence microscopy images of a triple epitope tagged BF *T. brucei* cell line expressing the SLAB components: mNG::SLAP1 (SLAP1-mNG, green), SNAP3::6HA (SNAP3-HA, magenta) and VEX1::12myc (VEX1-myc, red) after treatment with the Pol I transcription inhibitor BMH-21 for the time indicated in hours (h). Nuclei (dashed lines) are stained with DAPI (blue). Scale bar = 1 μm.
- **d)** Quantitation of the percentage (%) of cells in **c**) with either one or two SLAB foci (as monitored using mNG::SLAP1) after treatment with BMH-21 for the time indicated in hours (h). Values shown are the averages of three biological replicates with error bars indicating ± SD. P values were determined using a two tailed paired Student's t-test. *, P=0.0298.
- **e)** RNA-FISH imaging of the cell line shown in **c)** after incubation with BMH-21 for the time indicated in hours (h). RNA-FISH was performed to detect nascent transcript

from the active BES1 using a VSG pseudogene probe (VSGΨ, magenta). The nuclei are stained with DAPI (blue) with the scale bar = 1 μm.

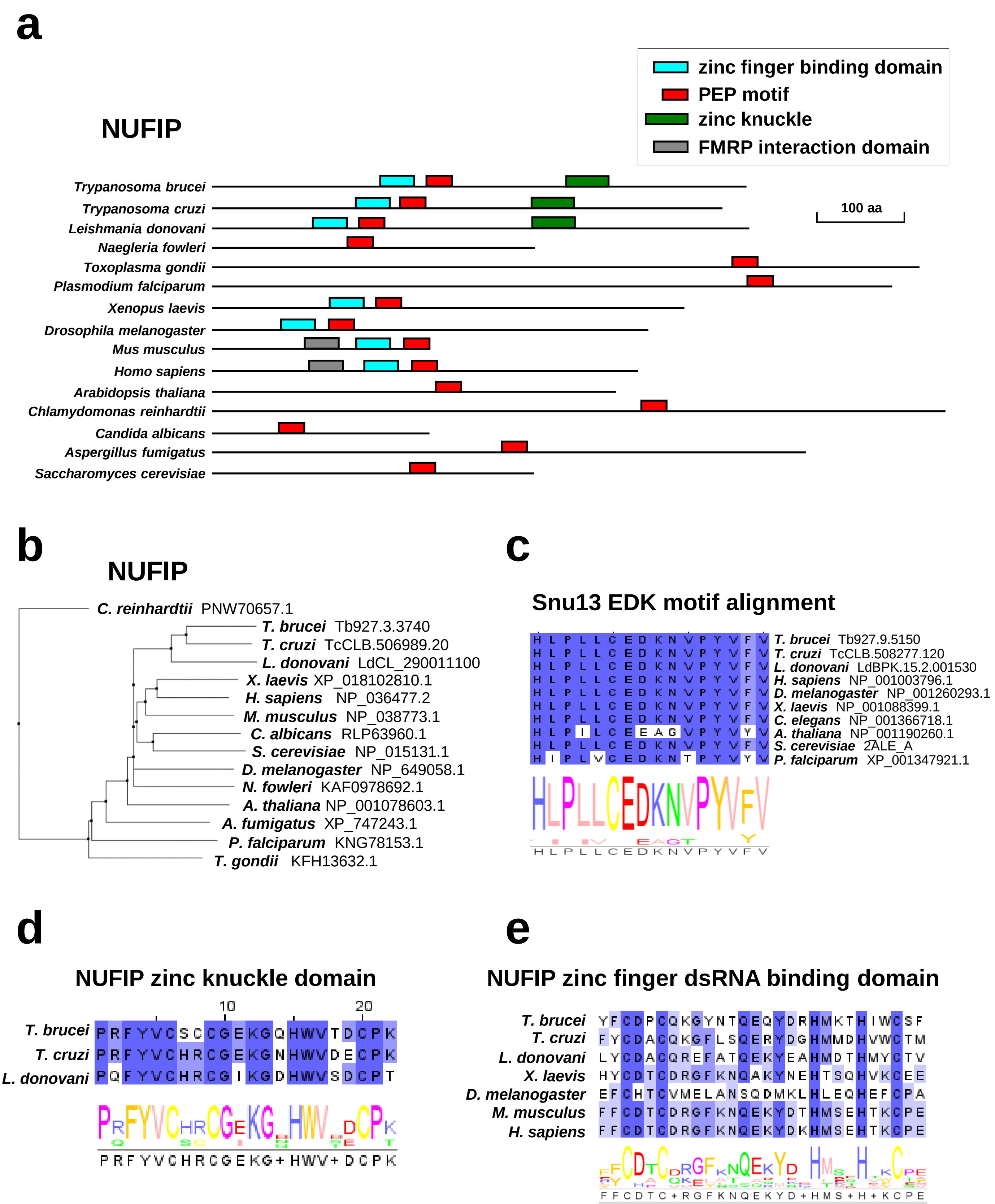
a
NUFIP
zinc finger binding domain
PEP motif
zinc knuckle
FMRP interaction domain
100 aa
Trypanosoma brucei
Trypanosoma cruzi
Leishmania donovani
Naegleria fowleri
Toxoplasma gondii
Plasmodium falciparum
Xenopus laevis
Drosophila melanogaster
Mus musculus
Homo sapiens
Arabidopsis thaliana
Chlamydomonas reinhardtii
Candida albicans
Aspergillus fumigatus
Saccharomyces cerevisiae
b
NUFIP
C. reinhardtii PNW70657.1
T. brucei Tb927.3.3740
T. cruzi TcCLB.506989.20
L. donovani LdCL_290011100
X. laevis XP_018102810.1
H. sapiens NP_036477.2
M. musculus NP_038773.1
C. albicans RLP63960.1
S. cerevisiae NP_015131.1
D. melanogaster NP_649058.1
N. fowleri KAF0978692.1
A. thaliana NP_001078603.1
A. fumigatus XP_747243.1
P. falciparum KNG78153.1
T. gondii KFH13632.1
c
Snu13 EDK motif alignment
T. brucei Tb927.9.5150
T. cruzi TcCLB.508277.120
L. donovani LdBPK.15.2.001530
H. sapiens NP_001003796.1
D. melanogaster NP_001260293.1
X. laevis NP_001088399.1
C. elegans NP_001366718.1
A. thaliana NP_001190260.1
S. cerevisiae 2ALE_A
P. falciparum XP_001347921.1
H L P L L C E D K N V P Y V F V
d
NUFIP zinc knuckle domain
10
20
T. brucei
T. cruzi
L. donovani
P R F Y V C H R C G E K G + H W V + D C P K
e
NUFIP zinc finger dsRNA binding domain
T. brucei
T. cruzi
L. donovani
X. laevis
D. melanogaster
M. musculus
H. sapiens
F F C D T C + R G F K N Q E K Y D + H M S + H + K C P E

Supplementary Figure 6

NUFIP is highly conserved across eukaryotes and Kinetoplastid NUFIP has a unique zinc knuckle domain.

a) Schematic of NUFIP homologues in different eukaryotes. Key protein domains and motifs are shown with coloured boxes. The PEP motif (red box) is conserved across all NUFIP homologues.

b) Phylogenetic tree of NUFIP homologues shown in **a**). The tree is drawn to scale, with branch lengths measured in number of substitutions per site. Accession numbers are indicated after the species name.

c) Alignments of the Snu13 EDK motif which binds the PEP motif of NUFIP. Conserved amino acid residues are highlighted. Darker shades indicate higher percentage identity across homologues. A position weight matrix of the alignment consensus sequence is shown underneath. Accession numbers are indicated after the species names.

d) As in **c)** only alignments of the NUFIP zinc knuckle domain are shown.

e) As in **c)** only alignments of the NUFIP zinc finger dsRNA binding domain are shown.

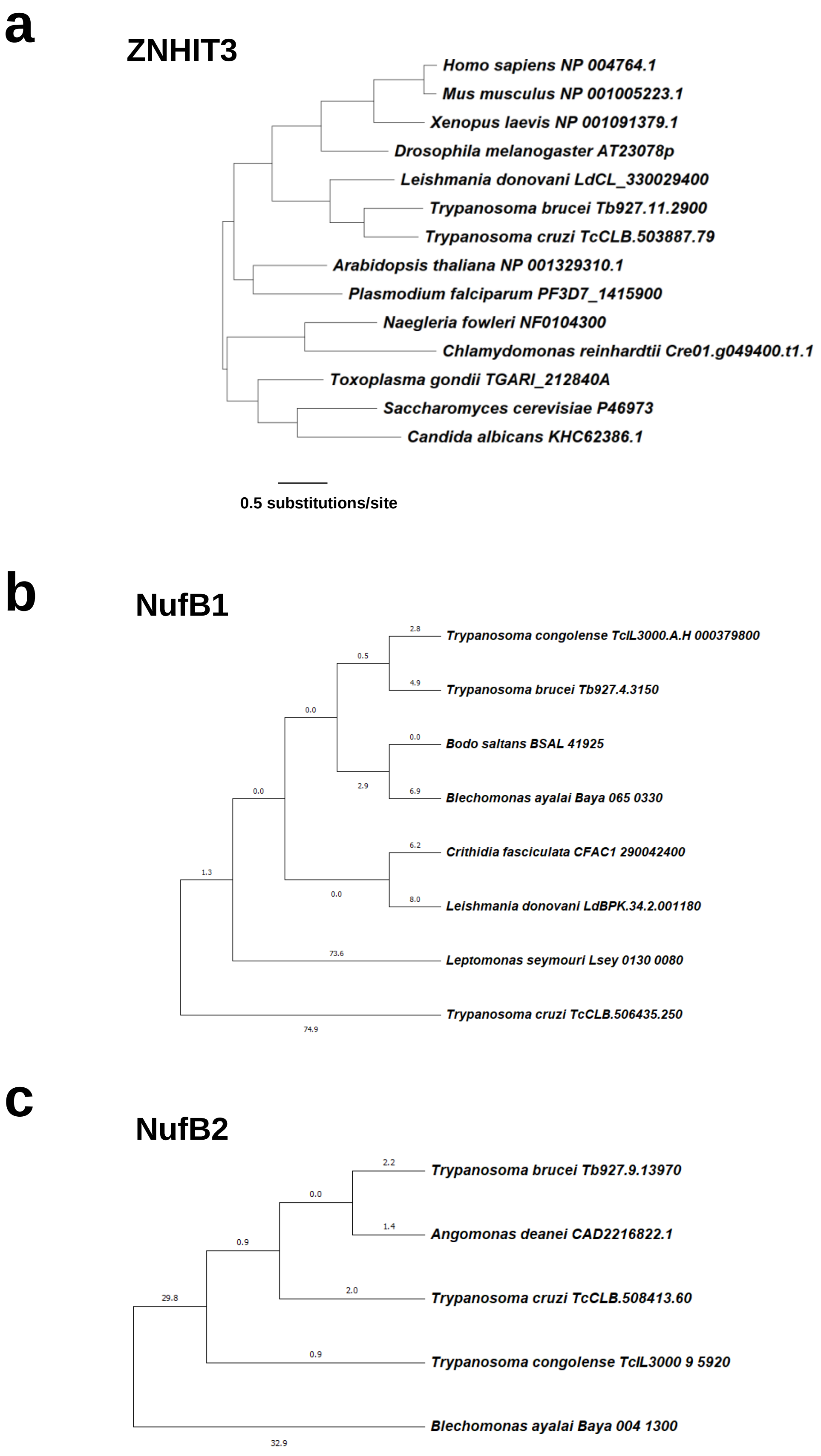
a
ZNHIT3
Homo sapiens NP 004764.1
Mus musculus NP 001005223.1
Xenopus laevis NP 001091379.1
Drosophila melanogaster AT23078p
Leishmania donovani LdCL_330029400
Trypanosoma brucei Tb927.11.2900
Trypanosoma cruzi TcCLB.503887.79
Arabidopsis thaliana NP 001329310.1
Plasmodium falciparum PF3D7_1415900
Naegleria fowleri NF0104300
Chlamydomonas reinhardtii Cre01.g049400.t1.1
Toxoplasma gondii TGARI_212840A
Saccharomyces cerevisiae P46973
Candida albicans KHC62386.1
0.5 substitutions/site
b
NufB1
Trypanosoma congolense TcIL3000.A.H 000379800
Trypanosoma brucei Tb927.4.3150
Bodo saltans BSAL 41925
Blechomonas ayalai Baya 065 0330
Crithidia fasciculata CFAC1 290042400
Leishmania donovani LdBPK.34.2.001180
Leptomonas seymouri Lsey 0130 0080
Trypanosoma cruzi TcCLB.506435.250
2.8
4.9
0.5
0.0
0.0
6.9
2.9
0.0
6.2
8.0
0.0
1.3
73.6
74.9
c
NufB2
Trypanosoma brucei Tb927.9.13970
Angomonas deanei CAD2216822.1
Trypanosoma cruzi TcCLB.508413.60
Trypanosoma congolense TcIL3000 9 5920
Blechomonas ayalai Baya 004 1300
2.2
0.0
1.4
0.9
2.0
29.8
0.9
32.9

Supplementary Figure 7

Conservation of NUFIP body components in Kinetoplastids

- **a)** Phylogenetic tree of ZNHIT3. The tree is drawn to scale with branch lengths measured in the number of substitutions per site.
- **b)** As in **a)** but analysis of NufB1
- **c)** As in **a**) but analysis of NufB2.

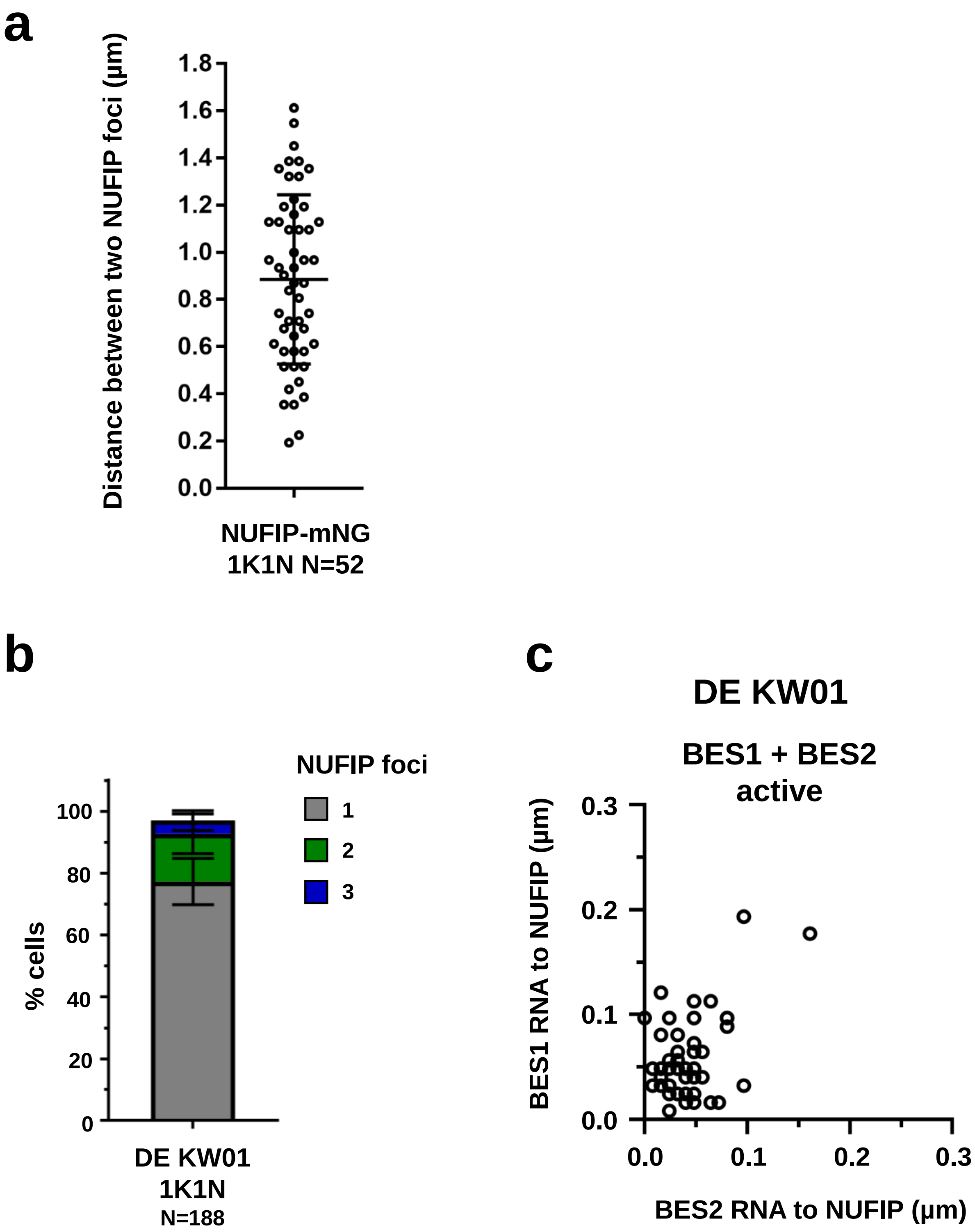
a
Distance between two NUFIP foci (μm)
1.8
1.6
1.4
1.2
1.0
0.8
0.6
0.4
0.2
0.0
NUFIP-mNG
1K1N N=52
b
% cells
100
80
60
40
20
0
NUFIP foci
1
2
3
DE KW01
1K1N
N=188
c
DE KW01
BES1 + BES2
active
BES1 RNA to NUFIP (μm)
0.3
0.2
0.1
0.0
0.0
0.1
0.2
0.3
BES2 RNA to NUFIP (μm)

Supplementary Figure 8

In double-expresser trypanosomes, the two unstably active ESs associate with a single NUFIP body.

a) Quantitation of the distance between NUFIP foci in cells where two NUFIP bodies were visible within the nucleus. Data were collected from two biological replicates with error bars indicating ± SD.

b) Quantitation of the distance between nascent RNA from BES1 or BES2 and the NUFIP body. This was done in the double expresser DE-KW01-MS2-V02 cell line shown in Fig. 5c, in which nascent RNA from BES1 or BES2 is detectable. N=46 from two biological replicates.

c) Quantitation of the percentage (%) of double-expresser DE KW01-MS2-V02 cells in G1 (1K1N) with different numbers of NUFIP foci (1-3) detectable, as determined by SR-SIM imaging of NUFIP::mNG. Two biological replicates were performed with error bars indicating ± SD.

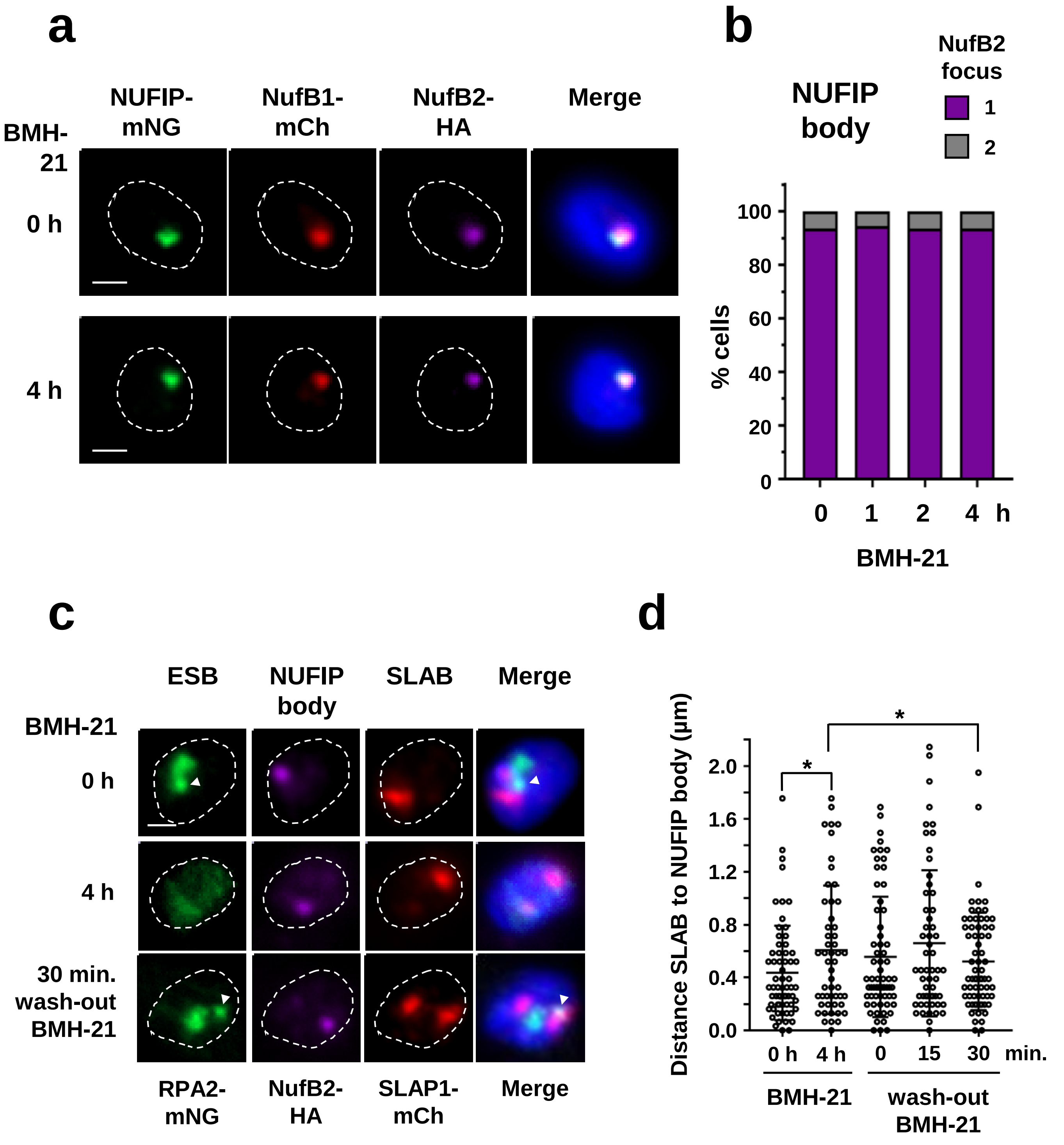

a
NUFIP-mNG
NufB1-mCh
NufB2-HA
Merge
BMH-21
0 h
4 h
b
NUFIP body
NufB2 focus
1
2
% cells
0
20
40
60
80
100
0
1
2
4 h
BMH-21
c
ESB
NUFIP body
SLAB
Merge
BMH-21
0 h
4 h
30 min. wash-out BMH-21
RPA2-mNG
NufB2-HA
SLAP1-mCh
Merge
d
Distance SLAB to NUFIP body (µm)
0.0
0.4
0.8
1.2
1.6
2.0
*
*
0 h
4 h
0
15
30
min.
BMH-21
wash-out BMH-21

Supplementary Figure 9

A single NUFIP body is present in most BF *T. brucei* cells, and is not disrupted by inhibition of RNA Pol I transcription.

a) Representative immunofluorescence microscopy images of triple epitope tagged BF *T. brucei* cell line which expresses the NUFIP body components: NUFIP::mNG (NUFIP-mNG, green), mCh::NufB1 (NufB1-mCh, red) and NufB2::6xHA (NufB2-HA, magenta). Pol I transcription was inhibited through treatment with 1 µM BMH-21 for the time indicated in hours (h). Nuclei (dashed lines) were stained with DAPI (blue). Scale bar = 1 µm.

b) Quantitation of the percentage (%) of cells containing different numbers of NUFIP bodies (as visualised using NufB2), after treatment with BMH-21 for the time indicated in hours (h). Values shown are the averages from two biological replicates with error bars indicating ± SD.

c) Immunofluorescence microscopy imaging of a triple epitope tagged cell line where the ESB, NUFIP and SLAB bodies are simultaneously visualised. This line expresses mNG::RPA2 (RPA2-mNG, green), NufB2::6xHA (NufB2-HA, magenta) and mCh::SLAP1 (SLAP1-mCh, red). A white arrowhead indicates the ESB. The cell line was incubated with the Pol I transcriptional inhibitor BMH-21 for the time indicated in hours (h). Pol I transcription was allowed to reinitiate for 30 minutes (min) after washout of the BMH-21 inhibitor.

d) Quantitation of distance between the SLAB (SLAP1) or NUFIP body (NufB2) using the cell line in **c)** after BMH-21 treatment for the time indicated in hours (h) or after wash-out of BMH-21 for the time indicated in minutes (min). N = minimally 52 G1 cells (1K1N) from two biological replicates. Error bars indicate ± SD. *, $P \leq 0.05$. P values were determined using a two tailed paired Student's t-test. For 0 h versus 4 h

BMH-21 treatment P=0.0438, for 4 h BMH-21 treatment versus 30 min BMH-21 washout P=0.0156.

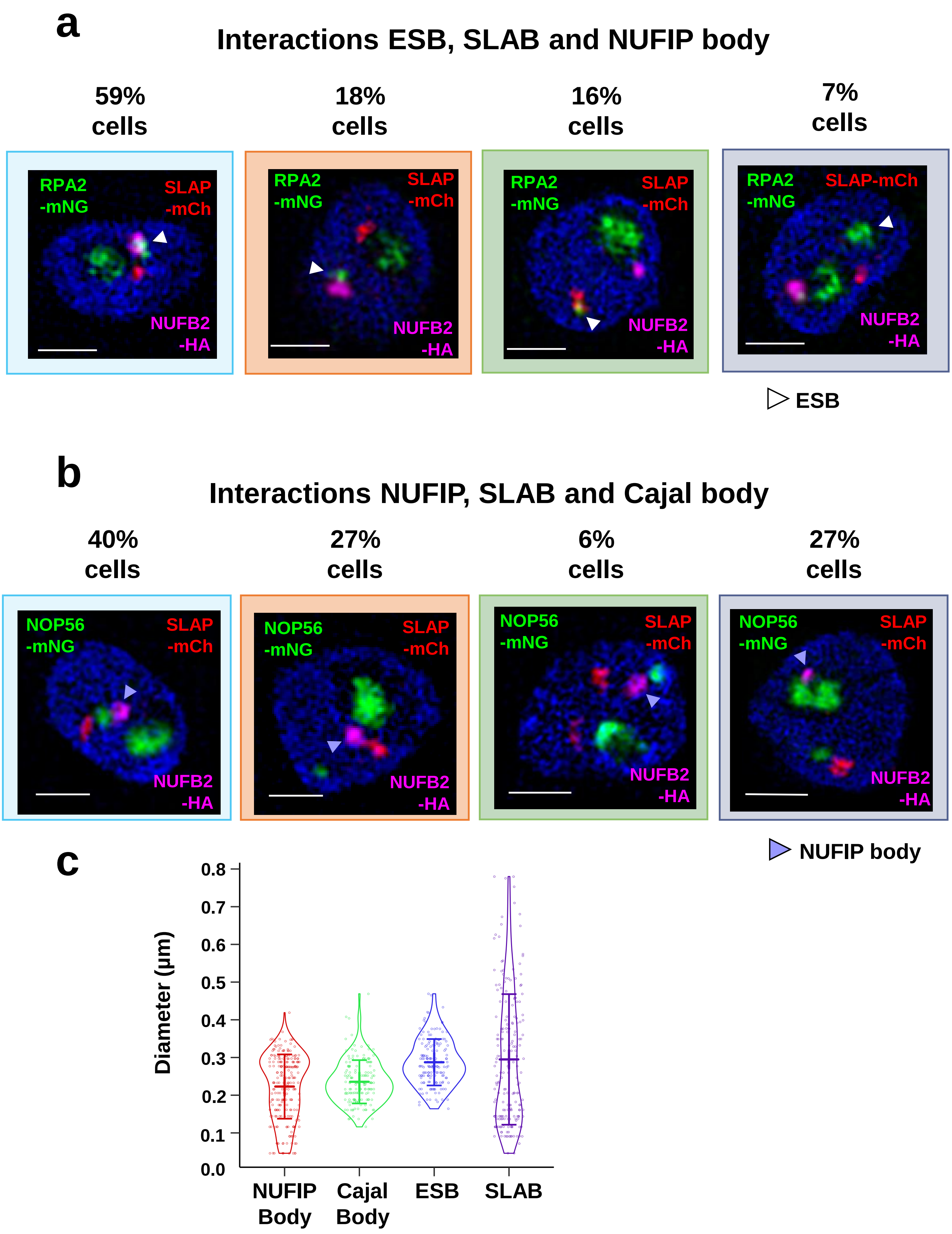
a
Interactions ESB, SLAB and NUFIP body
59% cells
18% cells
16% cells
7% cells
RPA2 -mNG
SLAP -mCh
NUFB2 -HA
SLAP-mCh
ESB
b
Interactions NUFIP, SLAB and Cajal body
40% cells
27% cells
6% cells
27% cells
NOP56 -mNG
SLAP -mCh
NUFB2 -HA
NUFIP body
c
Diameter (µm)
0.0
0.1
0.2
0.3
0.4
0.5
0.6
0.7
0.8
NUFIP Body
Cajal Body
ESB
SLAB

Supplementary Figure 10

An assembly of nuclear bodies associates with the active ES.

a) Preferential colocalisation of the ESB (RPA2-mNG, green) with the SLAB (SLAP-mCh, red) and NUFIP bodies (NufB2-HA, magenta) in BF *T. brucei.* Example microscopy images of the cells quantitated in the different coloured quadrants in Fig. 7b are shown. The nuclei are stained with DAPI (blue) and the ESB indicated with a white arrowhead. The percentage (%) of the population distributing within the different quadrants is indicated. Scale bar= 1 μm.

b) Colocalisation of the Cajal body (NOP56-mNG, green) with NUFIP (NufB2-HA, magenta) and the SLAB (SLAP-mCh, red) bodies in BF *T. brucei*. This is as described in **a)**, only microscopy examples of the cells quantitated in the different coloured quadrants in Fig. 7d are shown.

c) Quantitation of the diameters of the NUFIP body, Cajal body, Expression site body (ESB) and spliced leader array body (SLAB). This was determined using cell lines expressing mNG::NOP56, mNG::RPA2, NUFIP::mNG and mNG::SLAP respectively. Cells were analysed using SR-SIM imaging and data was collected from minimally 155 1K1N cells across two biological replicates. Error bars indicate ± SD.

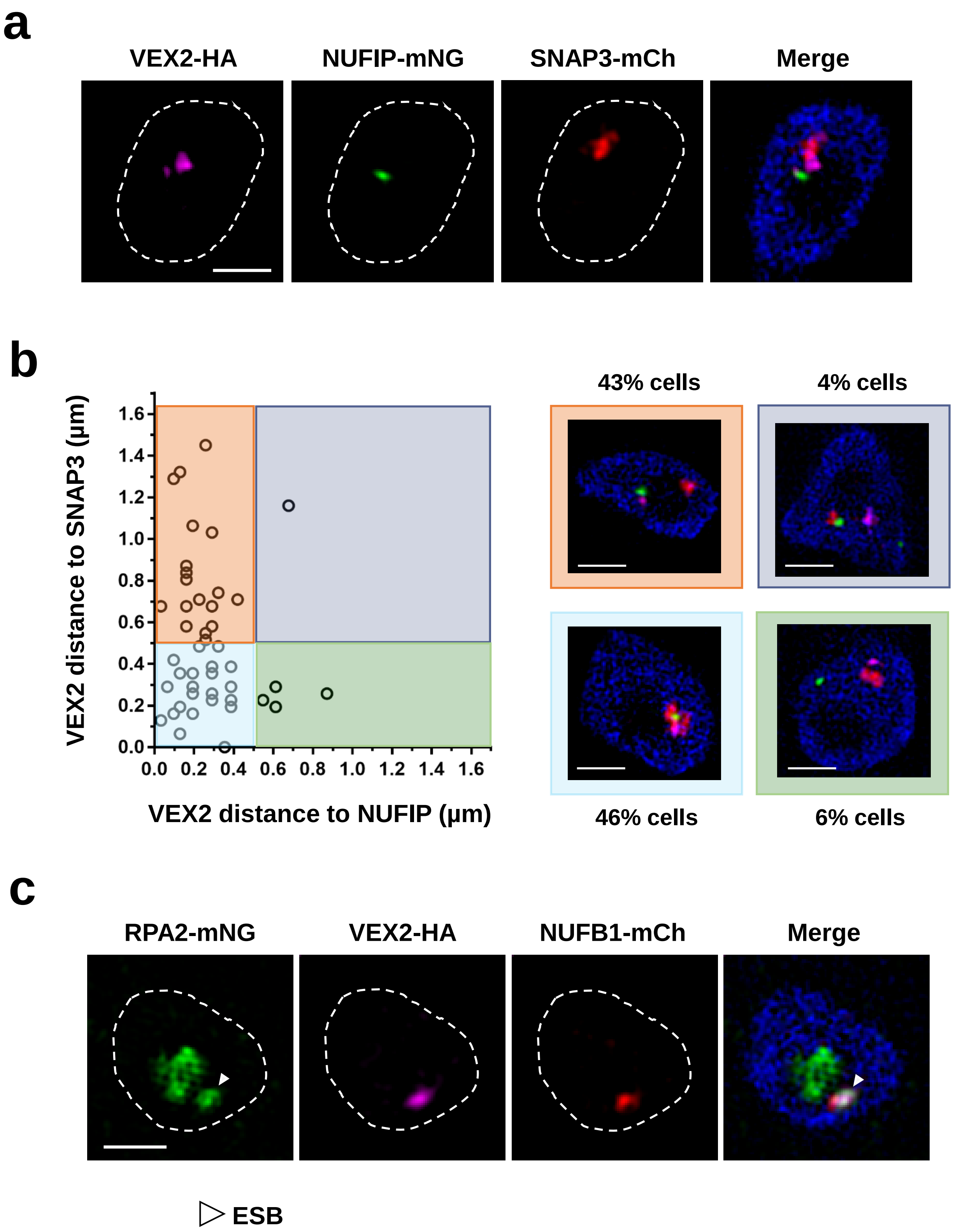

a
VEX2-HA
NUFIP-mNG
SNAP3-mCh
Merge
b
43% cells
4% cells
46% cells
6% cells
VEX2 distance to SNAP3 (µm)
VEX2 distance to NUFIP (µm)
0.0
0.2
0.4
0.6
0.8
1.0
1.2
1.4
1.6
c
RPA2-mNG
VEX2-HA
NUFB1-mCh
Merge
ESB

Supplementary Figure 11

The ESB is in close proximity to the NUFIP and the SLAB bodies.

- **a)** SR-SIM imaging of a triple epitope tagged BF *T. brucei* line expressing the ESB marker VEX2::6xHA (VEX2-HA, magenta), the NUFIP body marker NUFIP::mNG (NUFIP-mNG, green) and the SLAB marker SNAP3::mCh (SNAP3-mCh, red).
- **b)** Quantitation of the distance between the ESB (VEX2) to either the SLAB (SNAP3) or the NUFIP bodies. The coloured quadrants indicate whether the SLAB and NUFIP bodies are within 500 nm of the ESB. N=48 from two biological replicates. On the right are example microscopy images from cells corresponding to the different coloured quadrants as indicated with the coloured frames. The foci are from cells shown in **a)**. The percentage (%) of cells located within each coloured quadrant is indicated.
- **c)** SR-SIM imaging of a triple epitope tagged cell line expressing ESB markers mNG::RPA2 (RPA2-mNG, green) and VEX2::6HA (VEX2-HA, magenta) as well as the NUFIP body marker mCh::NufB1(NufB1-mCh, red). The ESB is indicated with a white arrowhead.

For all microscopy images the nucleus (dashed line) is stained with DAPI (blue). All scale bars= 1 µm.

Nuclear bodies in PF *T. brucei*

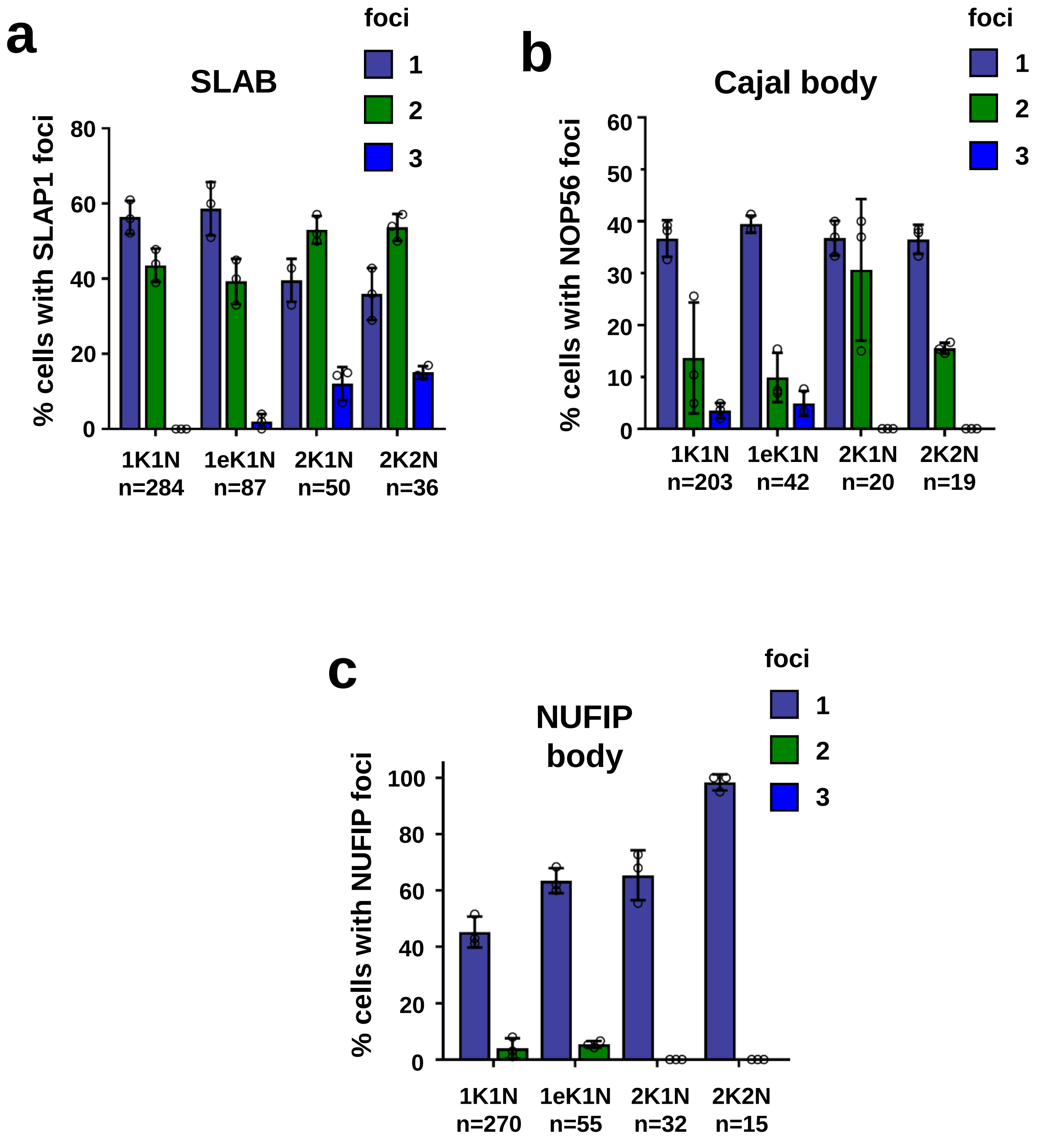

Supplementary Figure 12

The majority of PF *T. brucei* cells contain a single SLAB, NUFIP or Cajal body

- **a)** Quantitation of the percentage (%) of PF *T. brucei* cells with different numbers of SLAB foci, as visualised using fluorescence microscopy using cells expressing mNG::SLAP1. The number of foci (1-3) are indicated with coloured bars. Cells were monitored through the cell cycle, with cells with different numbers of kinetoplasts (K), elongated kinetoplasts (eK) or nuclei (N) indicated. The number (n) of cells counted is shown below. Values shown are from three biological replicates with error bars indicating ± SD.
- **b)** As in **a**), only the Cajal body was monitored using PF cells expressing mNG::NOP56.
- **c)** As in **a**), only the NUFIP body was monitored in PF cells expressing NUFIP::mNG.

Gene	mRNA molecules per BF cell, estimating VSG mRNA as 10% of 20,000 BF mRNAs (except procyclin)	mRNA half-life (min)	mRNA molecules produced for one half-life to maintain steady state levels	mRNA molecules produced for 60 min.	Number alleles (TREU927 genome assembly v5.1)	Single allele mRNA production rates (mRNA molecules produced for 60 min. from a single allele)
**VSG2**	2000	90	1000.0	666.7	1	666.7
**GPEET procyclin**	573	120	286.5	143.3	2	71.0
**EP procyclin**	1117	85	558.5	396.0	10	39.0
**alpha-tubulin**	396	77	197.8	153.6	8	19.2
**beta-tubulin**	293	66	146.4	133.4	8	16.7
**Glyceraldehyde 3-phosphate dehydrogenase, glycosomal**	175	120	87.3	43.6	4	10.9
**Elongation factor 1-alpha**	223	120	111.4	55.7	6	9.2
**Heat shock 70 kDa protein, mitochondrial precursor**	77	55	38.5	42.4	6	7.1
**Paraflagellar rod protein, PFR1**	59	49	29.7	36.6	10	3.7
**Histone H3, putative**	71	61	35.4	35.1	14	2.5
**Paraflagellar rod protein, PFR2**	44	67	22.0	19.8	10	2.0
**Typical Pol II transcribed gene**	1	12	0.5	2.5	2	1.3

Supplementary Table 1

List of mRNA copy number and mRNA half-life values used to calculate the mRNA production rate for VSG221 (BF), procyclin (PF) and selected Pol II transcribed genes (BF). The mRNA production rates shown were also adjusted to the number of alleles of the corresponding gene.

Gene ID	Gene product	mRNA molecules per BF cell, estimating VSG mRNA is 10% of 20,000 BF mRNAs	mRNA half-life in BF (min)	RNA molecules produced for one half life to maintain steady-state levels	mRNA production rate (mRNA molecules produced for 60 min)
**Tb427.BES40.22**	VSG221	2000	90	1000.0	666.7
**Tb427.BES40.2**	ESAG7	67	12	33.3	166.5
**Tb427.BES40.3**	ESAG6	57	12	28.7	143.4
**Tb427.BES40.4**	ESAG5 pseudogene	10	12	5.1	25.3
**Tb427.BES40.5**	ESAG3 pseudogene	20	12	10.2	50.9
**Tb427.BES40.6**	ESAG12	6	12	3.2	15.8
**Tb427.BES40.7**	ESAG4	2	12	0.8	4.0
**Tb427.BES40.8**	Hypothetical protein	25	12	12.4	61.8
**Tb427.BES40.9**	ESAG8	1	12	0.4	2.2
**Tb427.BES40.10**	ESAG3	22	12	11.2	56.0
**Tb427.BES40.11**	Hypothetical protein	5	12	2.7	13.5
**Tb427.BES40.12**	Hypothetical protein	4	12	2.2	11.2
**Tb427.BES40.13**	ESAG4	1	12	0.4	2.1
**Tb427.BES40.14**	ESAG8	2	12	1.1	5.4
**Tb427.BES40.15**	ESAG8	1	12	0.4	2.1
**Tb427.BES40.16**	ESAG3	15	12	7.6	37.8
**Tb427.BES40.17**	Hypothetical protein	6	12	2.8	13.8
**Tb427.BES40.18**	ESAG2	4	12	1.8	8.9
**Tb427.BES40.19**	ESAG11 pseudogene	1	12	0.3	1.5
**Tb427.BES40.20**	ESAG1	14	12	6.8	33.9

Supplementary Table 2

List of mRNA copy number and mRNA half-life values for VSG221 and ESAG mRNAs within the VSG221 ES (BES40) used to calculate mRNA production rates.

Proteins with one to four discrete nuclear foci identified using TrypTag		
**Gene ID**	**Description**	**Terminus tagged**
Tb927.4.3150	Hypothetical protein, conserved	N/ C
Tb927.3.3740	Zinc-finger double-stranded RNA binding, putative	C
Tb927.10.7070	Small nuclear RNA activating protein	N/ C
Tb927.11.3340	RNA binding protein 34, putative	C
Tb927.11.13380	ATP-dependent helicase, putative (VEX2)	C
Tb927.11.16920	VSG exclusion protein 1 (VEX1)	C
Tb927.9.13970	Hypothetical protein, conserved	N/ C
Tb927.11.2900	HIT zinc finger, putative	N/ C
Tb927.11.9950	Hypothetical protein, conserved	N/ C

Supplementary Table 3

Proteins with a Cajal body-like localisation identified using the TrypTag database.

Cell line	Parental cell line	Construct(s) transfected	Constructs/ cell line generated by
SM221PUR (S16)	N/A	N/A	Wirtz *et al*., 1999
mNG::RPA2	SM221PUR	pEnT5B-mNG::RPA2	This study
rDNA-24xMS2	SM221PUR	rDNA-bsr-24xMS2	This study
221ES-prom-24xMS2	SM221PUR	p221ES-bsr-24xMS2	This study
221ES-telo-24xMS2	SM221PUR	p70bp-24xMS2-221CTR	This study
tubulin-24xMS2	SM221PUR	pTub-bsr-24xMS2	Budzak *et al*., 2019
KW01-MS2-V02	KW01	pLew100V5-mCherry-eGFP-RNAi/ p70bp-bsr-V02-24xMS2	Budzak *et al*., 2019
mNG::SLAP1	SM221PUR	pEnT5B-mNG::SLAP1	This study
SNAP3::6xHA	SM221PUR	pEnT5B-SNAP3::6xHA	This study
VEX1::12xmyc	SM221PUR	pNATVEX1 ::12xmyc-blast	Construct: Glover *et al*., 2016<br>Cell line: This study
mNG::SLAP1-SNAP3::6xHA-VEX1::12xmyc	mNG::SLAP1	pNATVEX1::12xmyc-phleo/ pEnT5H-SNAP3::6xHA	This study
KW01-mNG::SLAP1	KW01-MS2-V02	pEnT5H-mNG::SLAP1	This study
mNG::SLAP1-SLAP1-RNAi	mNG::SLAP1	pDexV4-SLAP1-RNAi	This study
mNG::SLAP1-SLAP1-RNAi-SNAP3::6xHA	mNG::SLAP1-SLAP1-RNAi	pEnT5H-SNAP3::6xHA	This study
mNG::SLAP1-SLAP1-RNAi-TdT::RPA2	mNG::SLAP1-SLAP1-RNAi	pEnT5H-TdT::RPA2	This study
mNG::SLAP1-VEX1-RNAi	mNG::SLAP1	pDexV4-VEX1-RNAi	This study
NUFIP::mNG	SM221PUR	pEnT5B-NUFIP ::mNG	This study
NUFB2::mNG	SM221PUR	pEnT5B-NUFB2 ::mNG	This study
mNG::NUFB1	SM221PUR	pEnT5B-mNG::NUFB1	This study
mNG::NOP56-bsr	SM221PUR	pEnT5B-mNG::NOP56	This study
mCh::SLAP1-NUFIP::mNG	NUFIP::mNG	pEnT5H-mCh::SLAP1	This study
mNG::NOP56-3xHA-Fibrillarin2	SM221PUR	-pEnT5B-3xHA::Fibrillarin2<br>-pEnT5H-mNG::NOP56	This study
TdT::RPA2 mNG::NOP56	mNG::NOP56-bsr	pEnT5H-TdT::RPA2	This study

NUFIP::mNG-NUFB2::6xHA-mCh::NUFB1	NUFIP::mNG	-pEnT5Ph-NUFB2 ::6xHA<br>-pEnT5H-mCh ::NUFB1	This study
RBP34::mNG NUFB2::6xHA	SM221PUR	-pEnT5Ph-NUFB2 ::6xHA<br>-pEnT5B-RBP34 ::mNG	This study
NUFB2::6xHA-ZNHIT3::mNG	SM221PUR	-pEnT5Ph-NUFB2 ::6xHA<br>-pEnT5B-ZNHIT3::mNG	This study
V02-24xMS2	(V02+)PG_VHC	70bp-24xMS2-V02CTR	Budzak *et al.*, 2019
V02-24xMS2-NUFIP::mNG	V02-24xMS2	pEnT5H-NUFIP::mNG	This study
KW01-MS2-V02-NUFIP::mNG	KW01-MS2-V02	pEnT5H-NUFIP::mNG	This study
mNG::RPA2-VEX2::6xHA-mCh::NUFB1	mNG::RPA2	-pEnT5H-mCh ::NUFB1<br>-pEnT5Ph-VEX2 ::6xHA	This study
NUFIP::mNG-VEX2::6xHA-SNAP3::mCh	NUFIP::mNG	-pEnT5Ph-VEX2 ::6xHA<br>-pEnT5H-SNAP3::mCh	This study
mCh::SLAP1-NUFB2::6xHA-mNG::NOP56	mNG::NOP56-bsr	-pEnT5Ph-NUFB2 ::6xHA<br>-pEnT5H-mCh ::NUFB1	This study
mNG::RPA2-mCh::SLAP1-EXAP2::6xHA	mNG::RPA2	-pEnT5H-mCh ::SLAP1<br>-pEnT5Ph-NUFB2 ::6xHA	This study
p221ES-bsr-24xMS2 mNG::RPA2	p221ES-bsr-24xMS2	pENT5H-mNG::RPA2	This study
p221ES-bsr-24xMS2 Halo::RPA2	p221ES-bsr-24xMS2	pENT5H-Halo::RPA2	This study
pSmOx PCF	N/A	pSmOx	Poon *et al.*, 2012
pSmOx-EP1-24xMS2	pSmOx PCF	EP1-hyg-24xMS2	This study
pSmOx-EP1-24xMS2-NUFIP::mNG	pSmOx-EP1-24xMS2	pEnT5B-NUFIP:mNG	This study
pSmOx-EP1-24xMS2-mNG::SLAP1	pSmOx-EP1-24xMS2	pEnT5B-mNG::SLAP1	This study
pSmOx-mNG::SLAP1-SNAP3::6xHA-VEX1::12xmyc	pSmOx-PCF	pEnT5B-mNG::SLAP1<br>pEnT5H-SNAP3::6xHA<br>pNAT-VEX1::12xmyc	This study
pSmOx-NUFIP::mNG-NUFB2::6xHA	pSmOx-PCF	pEnT5B-NUFIP::mNG<br>pEnT5Ph-NUFB2 ::6xHA	This study
pSmOx-mNG::NOP56-3xHA-Fibrillarin2	pSmOx-PCF	pEnT5H-mNG::NOP56<br>pEnT5B-3xHA::Fibrillarin2	This study
pSmOx-mCh::SLAP1-NUFB2::6xHA-	pSmOx-PCF	-pEnT5N-mCh::SLAP1<br>-pEnT5Ph-NUFB2 ::6xHA	This study

Supplementary Table 4

Overview of all cell lines generated in this study. Tagging constructs using the pEnT5 vector were used with either hygromycin (pEnT5H), phleomycin (pEnT5Ph), blasticidin (pEnT5B) or neomycin (pEnT5N) drug selection markers.

Primers qPCR

qPCR target	Primer pair	Sequence 5'-3'
**Actin mRNA**	Actin_1031s	GTTCCATCCTCTCATCACTA
	Actin_1091as	TCGTATTCACTCTTCGTTATC
**VSG pseudogene**	Pseudo_221f	GAATGGGGAGAGTGAAACCA
	Pseudo_221r	TGCATTTGTTCCTGCAGTTC
**rRNA precursor**	70srRNA_up_182s	TTGAAGGGAATGCAAAAGTGTA
	70srRNA_up_68s	AACTGGAAGAGACGGAGGTAAA
**Actin precursor**	ActinPrec_qPCR_s	GGTTGTAGGCATCTTGTTTA
	ActinPrec_qPCR_as	AGACCTTGCTGTGCAATAC
**VSG221 ES promoter pre-mRNA**	Pre_221ESp_F1	TCCCGTGGTTCCTTCGTCCA
	Pre_221ESp_R1	CTCCACACAGCGGAAGAGGC
**VEX1 mRNA**	VEX1-qPCR_F2	AGGGAACGTGATCGCCTTTT
	VEX1-qPCR_R2	GCTTGGCTACGCAAATCTCG
**SLAP1 mRNA**	SLAP1-qPCR_F1	CCTCTGGACGATCAATGGGA
	SLAP1-qPCR_R1	AGTACCACTGCACAACTGGA

Primers for RNAi target fragments

RNAi target	Primer pair	Sequence 5'-3'
**VEX1 sense target fragment**	VEX1_s_f_HindIII	TGATCAAGCTTCCGAATTTAAGCATGCGATG
	VEX1_s_r_AscI	TGATCGGCGCGCCCGAAGCAGGGTGAAGTGATA
**VEX1 anti-sense target fragment**	VEX1_as_f_BamHI	TGATCGGATCCCCGAATTTAAGCATGCGATG
	VEX1_as_r_XbaI	TGATCTCTAGACGAAGCAGGGTGAAGTGATA
**SLAP1 sense target fragment**	SLAP1_s_f_HindIII	ATTTATAAGCTTCAGTAAATGAGCAGCGAC
	SLAP1_s_r_AscI	ATTTATGGCGCGCCCACCGCTGCGCCTCGGGC
**SLAP1 anti-sense target fragment**	SLAP1_as_f_BamHI	ATTTATGGATCCCAGTAAATGAGCAGCGAC
	SLAP1_as_r_XbaI	ATTTATTCTAGACACCGCTGCGCCTCGGGC

Supplementary Table 5

PCR primers used in this study.

RNA-FISH probes

RNA-FISH target	Probe name	Sequence 5’-3’
**U2 snRNA**	U2 snRNA_1	TCTTAGCTAAATAGCCGAGA
	U2 snRNA_2	CGTATCAGGAGTTACTCTGA
	U2 snRNA_3	TTTTGATCCTTGGGCCAAAG
	U2 snRNA_4	AAGAACGCGGGACAGCCAAC
	U2 snRNA_5	TCCATCCGGACAAGTGGAAC
**SL-RNA intron**	SL RNA-1	CTACTGGGAGCTTCTCATAC
	SL RNA-2	CACAAATGCGTGTGTTGGCC
	SL RNA-3	AGTATGCGGCGGGAACCAAC
	SL RNA-4	CGACCCCACCTTCCAGATTC

Supplementary Table 6

RNA-FISH probes used in this study.
