# Supplementary material for: An assembly of nuclear bodies associates with the active VSG expression site in African trypanosomes
Source: Nat Commun. 2022 Jan 10;13:101. doi: 10.1038/s41467-021-27625-6 (PMC8748868; doi:10.1038/s41467-021-27625-6)
Supplement: Supplementary file 3 — Reporting Summary [file 41467_2021_27625_MOESM3_ESM.pdf]

nature portfolio

Corresponding author(s): Professor Gloria Rudenko

Last updated by author(s): 08/11/2021

Reporting Summary

Nature Portfolio wishes to improve the reproducibility of the work that we publish. This form provides structure for consistency and transparency in reporting. For further information on Nature Portfolio policies, see our Editorial Policies and the Editorial Policy Checklist.

Statistics

For all statistical analyses, confirm that the following items are present in the figure legend, table legend, main text, or Methods section.

n/a	Confirmed	
☐	☒	The exact sample size (*n*) for each experimental group/condition, given as a discrete number and unit of measurement
☐	☒	A statement on whether measurements were taken from distinct samples or whether the same sample was measured repeatedly
☐	☒	The statistical test(s) used AND whether they are one- or two-sided<br>*Only common tests should be described solely by name; describe more complex techniques in the Methods section.*
☒	☐	A description of all covariates tested
☒	☐	A description of any assumptions or corrections, such as tests of normality and adjustment for multiple comparisons
☐	☒	A full description of the statistical parameters including central tendency (e.g. means) or other basic estimates (e.g. regression coefficient) AND variation (e.g. standard deviation) or associated estimates of uncertainty (e.g. confidence intervals)
☐	☒	For null hypothesis testing, the test statistic (e.g. *F*, *t*, *r*) with confidence intervals, effect sizes, degrees of freedom and *P* value noted<br>*Give P values as exact values whenever suitable.*
☒	☐	For Bayesian analysis, information on the choice of priors and Markov chain Monte Carlo settings
☒	☐	For hierarchical and complex designs, identification of the appropriate level for tests and full reporting of outcomes
☒	☐	Estimates of effect sizes (e.g. Cohen's *d*, Pearson's *r*), indicating how they were calculated

Our web collection on statistics for biologists contains articles on many of the points above.

Software and code

Policy information about availability of computer code

Data collection	For SR-SIM data collection, Zeiss Zen Black version 14.0.9.201 was used. For fluorescence microscopy data collection, AxioVision version 4.5 or Zeiss Zen Blue 2.5 pro were used.
Data analysis	Analyses were performed using R version 4.1.0, ImageJ (FIJI) version 1.52/1.53, GraphPad version 7.05, MEGA-X version 10.1.8, Zeiss Zen Black version 14.0.9.201, SnapGene version 4.3.2, Jalview version 2.11.1.0, PFAM (http://pfam.xfam.org/), PSI-BLAST (https://blast.ncbi.nlm.nih.gov/Blast.cgi?PAGE_TYPE=BlastSearch&PROGRAM=blastp&BLAST_PROGRAMS=psiBlast) and PONDR (http://www.pondr.com/)

For manuscripts utilizing custom algorithms or software that are central to the research but not yet described in published literature, software must be made available to editors and reviewers. We strongly encourage code deposition in a community repository (e.g. GitHub). See the Nature Portfolio guidelines for submitting code & software for further information.

Data

Policy information about availability of data

All manuscripts must include a data availability statement. This statement should provide the following information, where applicable:

- Accession codes, unique identifiers, or web links for publicly available datasets
- A description of any restrictions on data availability
- For clinical datasets or third party data, please ensure that the statement adheres to our policy

The datasets generated and analysed during the current study are available from the corresponding author on reasonable request. The TrypTag database (http://tryptag.org/) is a publicly available dataset which was used to screen protein localisation in T. brucei. The TrypTag database was accessed through TryTrypDB release 44 (https://tritrypdb.org/tritrypdb/app).

March 2021

Field-specific reporting

Please select the one below that is the best fit for your research. If you are not sure, read the appropriate sections before making your selection.

- [x] Life sciences
- [ ] Behavioural & social sciences
- [ ] Ecological, evolutionary & environmental sciences

For a reference copy of the document with all sections, see nature.com/documents/nr-reporting-summary-flat.pdf

Life sciences study design

All studies must disclose on these points even when the disclosure is negative.

Sample size	All experiments show results with mainly three and sometimes two biological replicates, the details of which are specified in the text. No statistical methods were used to predetermine sample size. The size of the samples was determined when sufficient enough to allow us to establish biological variability between samples.
Data exclusions	No data were excluded from the analyses.
Replication	All experimental replicates were successful and were performed in biological triplicate or biological duplicates which is specified in the figure legends. N values for each experiment are specified in the figure legends or the Source data file.
Randomization	For microscopy experiments, fields of view were selected at random and then all cells from a particular cell cycle stage, or across all cell cycle stages, were analysed from each field. All other experiments (such as qPCR) did not require randomisation as we were analysing a single highly controlled variable.
Blinding	We did not use blinding as we did not have predetermined views regarding what the results would be. In addition, all experiments were carried out independently minimally two or three times. Furthermore, some data were analysed independently by two seperate researchers.

Reporting for specific materials, systems and methods

We require information from authors about some types of materials, experimental systems and methods used in many studies. Here, indicate whether each material, system or method listed is relevant to your study. If you are not sure if a list item applies to your research, read the appropriate section before selecting a response.

Materials & experimental systems

n/a	Involved in the study	
[ ]	[x]	Antibodies
[ ]	[x]	Eukaryotic cell lines
[x]	[ ]	Palaeontology and archaeology
[x]	[ ]	Animals and other organisms
[x]	[ ]	Human research participants
[x]	[ ]	Clinical data
[x]	[ ]	Dual use research of concern

Methods

n/a	Involved in the study	
[x]	[ ]	ChIP-seq
[x]	[ ]	Flow cytometry
[x]	[ ]	MRI-based neuroimaging

Antibodies

Antibodies used	Non-commercially available primary antibodies:<br>Mouse anti-L1C6, kind gift of Prof. Keith Gull laboratory, Sir Dunn School of Pathology, University of Oxford.<br>Mouse anti-SUMO, kind gift of Prof. Miguel Navarro, IPBLN-CSIC, Granada.<br>Commercially available primary antibodies:<br>Rabbit anti-mCherry, Abcam, Cat# ab167453, Lot# GR3213077<br>Rabbit anti-myc, Abcam, Cat# ab9106, Lot# GR171077<br>Rabbit anti-HA, Abcam, Cat# ab18181, Lot# GR323668-3<br>Commercially available secondary antibodies:<br>Goat anti-mouse AlexaFluor647, Invitrogen, Cat# A21236, Lot# 2069817<br>Goat anti-rabbit AlexaFluor594, Invitrogen, Cat# A11037, Lot# 2079421<br>Goat anti-mouse AlexaFluor488, Invitrogen, Cat# A11001, Lot# 2247988<br>Goat anti-mouse Dylight594, Invitrogen, Cat# 35510, Lot# SG253603<br><br>All antibody dilutions are specified in the methods section.
Validation	Validation of non-commercially available primary antibodies:<br>Mouse anti-L1C6, validation available in Devaux et al., 2007, DOI: 10.1091/mbc.E06-09-0841<br>Mouse anti-SUMO, validation available in Lopez-Farfan et al., 2014, DOI: 10.1371/journal.ppat.1004545

Validation of all commercial primary and secondary antibodies for immunofluorescence microscopy is publicly available on the manufacturer's website:
Rabbit anti-mCherry: https://www.abcam.com/mcherry-antibody-ab167453.html
Rabbit anti-myc: https://www.abcam.com/myc-tag-antibody-ab9106.html
Rabbit anti-HA: https://www.abcam.com/ha-tag-antibody-hac5-ab18181.html
Goat anti-mouse AlexaFluor647: https://www.thermofisher.com/antibody/product/Goat-anti-Mouse-IgG-H-L-Highly-Cross-Adsorbed-Secondary-Antibody-Polyclonal/A-21236
Goat anti-rabbit AlexaFluor594: https://www.thermofisher.com/antibody/product/Goat-anti-Rabbit-IgG-H-L-Highly-Cross-Adsorbed-Secondary-Antibody-Polyclonal/A-11037
Goat anti-mouse AlexaFluor488: https://www.thermofisher.com/antibody/product/Goat-anti-Mouse-IgG-H-L-Cross-Adsorbed-Secondary-Antibody-Polyclonal/A-11001
Goat anti-mouse DyLight594: https://www.thermofisher.com/antibody/product/Goat-anti-Mouse-IgG-H-L-Secondary-Antibody-Polyclonal/35510
All commercially available primary antibodies were validated independently by immunofluorescence microscopy using cell lines not expressing the antigen of interest. All secondary antibodies were validated using samples not incubated with primary antibodies.

Eukaryotic cell lines

Policy information about cell lines

Cell line source(s)	Trypanosoma brucei brucei 427
Authentication	This cell line expresses single copy genes (i.e. VSG221) which allows its validation.
Mycoplasma contamination	Our cell lines were tested for mycoplasma and were found to be negative.
Commonly misidentified lines (See ICLAC register)	We are not using any other trypanosome strains in the laboratory.
